# The complete chloroplast genome sequence of *Ficus altissima* (Moraceae)

**DOI:** 10.1080/23802359.2020.1863874

**Published:** 2021-02-03

**Authors:** Yuan Zhang, Ruli Zhang, Chongli Deng, Junwen Yang, Guobin Deng

**Affiliations:** a Yunnan Academy of Biodiversity, Southwest Forestry University, Kunming, China; b College of Biodiversity Conservation, Southwest Forestry University, Kunming, China; c College of Forestry, Southwest Forestry University, Kunming, China

**Keywords:** Ficus, chloroplast genome, phylogenomic, Moraceae

## Abstract

*Ficus altissima* plays an important role on biodiversity in tropical forests. In this study, the complete chloroplast genome sequence and the genome features of *F. altissima* were analyzed using the Illumina NovaSeq platform. The whole chloroplast genome sequence of *F. altissima* is 160,251 including a large single-copy region (LSC, 88,468 bp), a small single-copy region (SSC, 20,009 bp), and a pair of repeat regions (IRs, 25,887 bp, each). Further gene annotation revealed the chloroplast genome contains 124 genes, including 79 protein-coding genes, 37 tRNA genes, and 8 rRNA genes. A total of 82 simple sequence repeats (SSRs) were identified in the chloroplast genome. Phylogenetic development was analyzed based on *F. altissima* with other species of Moraceae. This information will be useful for study on the evolution and genetic diversity of *F. altissima* in the future.

It is widely accepted that tropical rainforests support the richest biodiversity among terrestrial ecosystems. However, they are suffering from rapid loss of biodiversity due to lots of reasons, such as human disturbance and climate change (Ghazoul and Sheil 2010). As a keystone resource in tropical forests, *Ficus* L. constitutes the most distinctive of the widespread genera in tropical area since figs (*syconium*) have a complex obligatory mutualism with their pollinating agaonid fig wasps (Weiblen 2002; Dunn 2020), and figs are found in almost all tropical habitat types and geographic locations (Janzen 1979). Furthermore, *Ficus* as a genus comprising more than 800 species, also playing a critical role in biodiversity conservation in the tropics, because *Ficus* are the most important group of plants for fruit-eating vertebrates (Shanahan et al. 2001).

*Ficus altissima* Blume (subgenus *Urostigma*) is a monoecious fig tree species distributed across Asia (Berg and Corner 2005). It occurs naturally in tropical forests at Xishuangbanna, Yunnan, China. *F. altissima* is also frequently planted in cities and villages or near temples as an ornamental or sacred plant. The aerial root and branch of *F. altissima* can be used as traditional Chinese medicine, and tender leaves are also used as edible vegetable by Xishuangbanna local people. In recent years, the ecology of *F. altissima* has intrigued the interests of ecologists (Peng et al. 2008, 2010; Zhang et al. 2014), while the complete chloroplast genome has not been sequenced. Considering the chloroplast DNA-based studies can provide invaluable data for studying genetic history and phylogeny, and can also provide important information in designing conservation strategies for the species, in this study, we sampled the *F. altissima* from the Xishuangbanna tropical botanical garden, Chinese Academy of Science (21°55′ N, 101°15′ E, 555 m above sea level). A voucher specimen (YAB 202,005) was deposited at Yunnan Academy of Biodiversity, Southwest Forestry University, Yunnan, China. Then we sequenced, assembled and annotated the accurate chloroplast genome with the next-generation sequencing method to reveal the phylogenetic relationship of *F. altissima*.

For this study, the total genomic DNA of *F. altissima* was extracted from fresh leaves according to the modified CTAB methods described by Doyle and Doyle (1987). A genomic shotgun library with an insertion size of 340 bp was constructed. The libraries were constructed using standard protocols (NEB Next Ultra II DNA Library Prep Kit for Illumina), and sequenced on Illumina NovaSeq platform at Personalbio Biotech (Shanghai, China). We assembled the chloroplast genome using GetOrganelle software version 1.7.1 (Jin et al. 2020), and the assembled chloroplast genome was annotated through the online program CPGAVAS 2 (Shi et al. 2019) with *F. religiosa* chloroplast genome (GenBank accession number: NC_033979) as a reference, and assisted with manual correction. The raw sequencing reads used in this study has been deposited in SRA with the accession number SRR12665077 and the annotated chloroplast genome sequence has been deposited into the GenBank with the accession number MW013819.

The complete chloroplast genome of *F. altissima* was 160,251 bp in length, consisting of a large single copy region (LSC, 88,468 bp), a small single copy region (SSC, 20,009 bp), and two inverted repeat regions (IRa and IRb, 25, 887 bp). The total GC content was 36.92%, with IR regions (42.60%) higher than that in LSC (33.56%) and SSC regions (28.9%). The chloroplast genome of *F. altissima* contains a total of 124 genes, including 79 protein-coding genes, 8 rRNA genes, and 37 tRNA genes. Furthermore, a total of 82 SSR markers ranging from mononucleotide to pentanucleotide repeat motif were identified in *F. altissima* chloroplast genome.

To determine the phylogenetic relationship of *F. altissima*, based on complete chloroplast genomes of 25 species within the family Moraceae, with *Debregeasia orientalis* and *Boehmeria nivea* var. *nipononivea* as outgroup (Figure 1), chloroplast genomes were downloaded from NCBI. All chloroplast genomes were aligned using the program MAFFT v7.471 (Rozewicki et al. 2019), and phylogenetic tree (maximum likelihood) constructed by Iqtree software version 1.6.12 (Minh et al. 2020) with 1000 bootstrap replicates, best-fitted model has been confirmed is TVM + F+R2 by ModelTest-NG (Darriba et al. 2020). The phylogenetic analysis revealed that *F. altissima* closely clustered with *F. religiosa*. The complete chloroplast genome of *F. altissima* will provide essential data for future research on the phylogenetic and evolutionary relationship in genus of *Ficus*.

**Figure 1.**
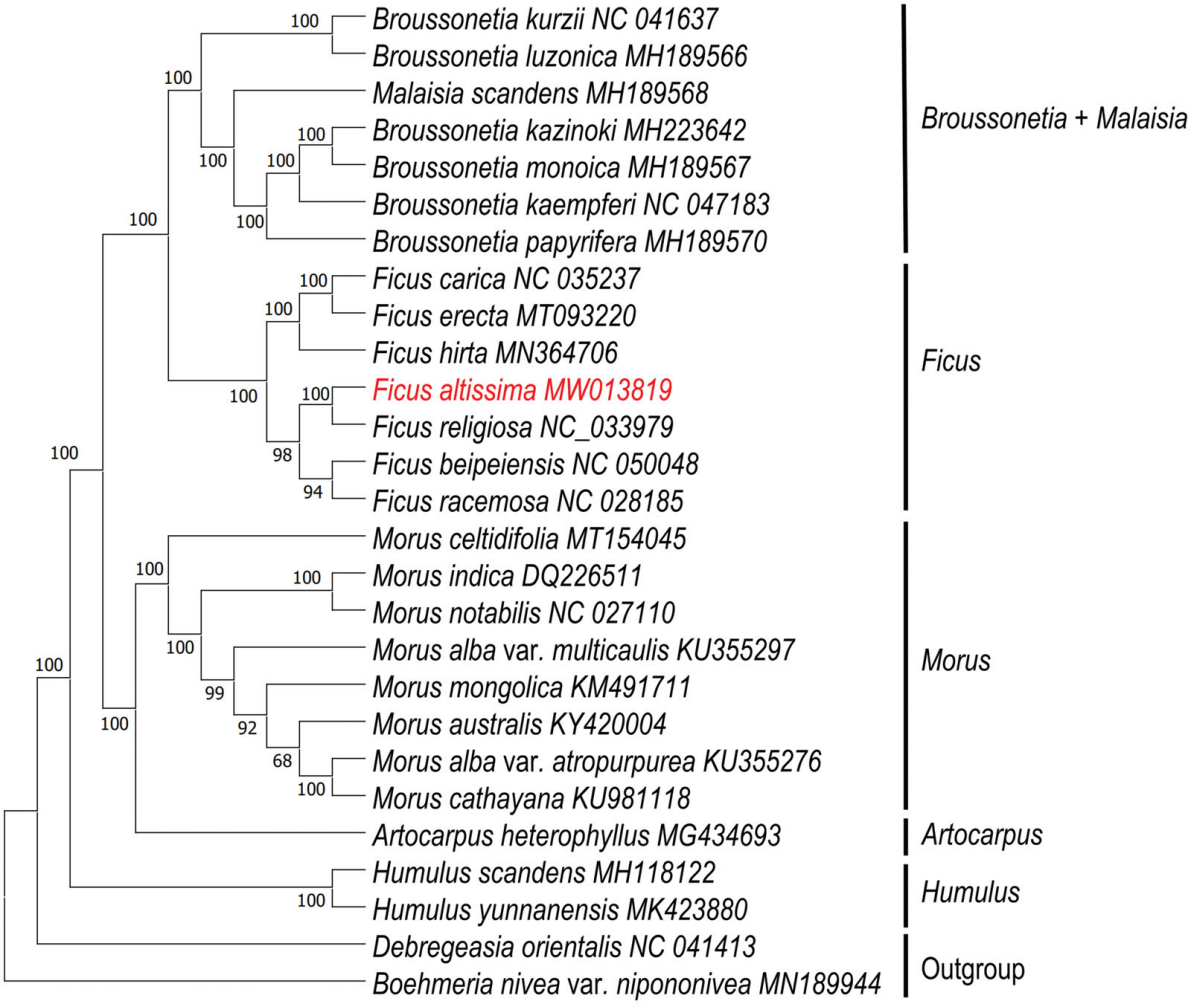
Maximum likelihood (ML) phylogenetic tree based on complete chloroplast genomes of 25 species of Moraceae, with Debregeasia orientalis and Boehmeria nivea var. nipononivea as outgroup. Numbers at nodes represent bootstrap values.

## Data Availability

The data that support the analyses and results of this study are openly available in Genbank with accession (MW013819) (https://www.ncbi.nlm.nih.gov/). Raw sequencing reads was deposited in SRA with BioProject accession (PRJNA664225). (https://www.ncbi.nlm.nih.gov/Traces/study/?acc=PRJNA664225).
